# Orthostatic Hypotension in Parkinson's Disease: Do Height and Weight Matter?

**DOI:** 10.1002/mds.28768

**Published:** 2021-08-23

**Authors:** Nicole Campese, Georg Goebel, Fabian Leys, Jean Pierre Ndayisaba, Sabine Eschlboeck, Christine Eckhardt, Cecilia Raccagni, Roberta Granata, Roberto Ceravolo, Stefan Kiechl, Klaus Seppi, Werner Poewe, Gregor K. Wenning, Alessandra Fanciulli

**Affiliations:** ^1^ Neurology Unit, Department of Clinical and Experimental Medicine University of Pisa Pisa Italy; ^2^ Department of Neurology Medical University of Innsbruck Innsbruck Austria; ^3^ Department of Medical Statistics, Informatics and Health Economics Medical University of Innsbruck Innsbruck Austria; ^4^ Department of Neurology Regional General Hospital Bolzano Bolzano Italy

**Keywords:** Parkinson's disease, orthostatic hypotension, body mass index, height

## Author Roles

1. Research project: A. Conception, B. Organization, C. Execution;

2. Statistical Analysis: A. Design, B. Execution, C. Review and Critique;

3. Manuscript: A. Writing of the first draft, B. Review and Critique.

N.C.: 1B, 1C, 2B, 3A

G.G.: 2A, 2B, 2C, 3B

F.L.: 1C, 3B

J.P.N.: 1B, 2C, 3B

S.E.: 1C, 3B

C.E.: 1C, 3B

C.R.: 1C, 3B

R.G.: 1B, 1C, 3B

R.C.: 1A, 3B

S.K.: 1A, 2C, 3B

K.S.: 1A, 1B, 3B

W.P.: 1A, 3B

G.K.W.: 1A, 1B, 2C, 3B

A.F.: 1A, 1B, 1C, 2A, 2B, 3B

## Financial Disclosures

Nicole Campese: nothing to report.

Georg Goebel: nothing to report.

Fabian Leys: nothing to report.

Jean Pierre Ndayisaba: nothing to report.

Sabine Eschlboeck: nothing to report.

Christine Eckhardt: nothing to report.

Cecilia Raccagni: nothing to report.

Roberta Granata: nothing to report.

Roberto Ceravolo: reports speaker fees from UCB Pharma, Zambon, General Electric, Lusofarmaco, AbbVie and Bial, outside of the submitted work.

Stefan Kiechl: reports support from the Austrian Research Promotion Agency FFG, outside of the submitted work.

Klaus Seppi: reports personal fees from Teva, UCB, Lundbeck, AOP Orphan Pharmaceuticals AG, Roche, Grünenthal, and AbbVie; honoraria from the International Parkinson and Movement Disorders Society; research grants from the FWF Austrian Science Fund, The Michael J. Fox Foundation, and International Parkinson and Movement Disorder Society, outside of the submitted work.

Werner Poewe: reports personal fees from: Alterity, AbbVie, Affiris, AstraZeneca, BIAL, Biogen, Britannia, Lilly, Lundbeck, Neuroderm, Neurocrine, Denali Pharmaceuticals, Novartis, Orion Pharma, Roche, Takeda, Teva, UCB and Zambon (consultancy and lecture fees in relation to clinical drug development programmes for PD); royalties: Thieme, Wiley Blackwell, Oxford University Press and Cambdridge University Press; grant support: MJFF, EU FP7 & Horizon 2020.

Gregor K. Wenning: reports consultancy and lecture fees from AbbVie, Affiris, AstraZeneca, Biogen, Lundbeck, Merz, Novartis, Ono, Teva, and Theravance, and research grants from the FWF Austrian Science Fund, the Austrian National Bank, the US MSA Coalition, Parkinson Fonds Austria, and International Parkinson and Movement Disorder Society, outside of the submitted work.

Alessandra Fanciulli: reports royalties from Springer Nature Publishing Group and Thieme Verlag; speaker's fees and honoraria from International Parkinson Disease and Movement Disorders Society, IOS Press, Impact Medicom, AbbVie, and Theravance Biopharma and research grants from the Stichting ParkinsonFond, US MSA Coalition, Dr Johannes Tuba Stiftung, and the Österreichischer Austausch Dienst, outside of the submitted work.

Every third person with Parkinson's disease (PD) may suffer from orthostatic hypotension (OH).^1^ Besides classic OH (cOH), transient orthostatic blood pressure (BP) drops may occur within the first minute upon standing, qualifying for transient OH (tOH).^2^ It is unclear whether morphometric factors, such as height and body mass index (BMI),^3^ promote OH in people with PD.

For this reason, we analyzed a previously published cohort of 173 European patients with PD for differences in height and BMI across individuals with laboratory‐confirmed cOH, tOH, or no OH.^2^

After comparing the morphometric and other clinicodemographic characteristics across patients with and without OH, we tested the association between BMI, height, and cOH or tOH, by calculating the area under the receiver operating characteristic (ROC) curve in males and females separately. The Youden index applied to the coordinates of the ROC curves determined the most accurate BMI and height cut‐offs distinguishing patients with either cOH or tOH from those without. Whenever significant cut‐offs were found, we compared the derived subgroups for differences in clinicodemographic features and autonomic function indices by means of univariate, binary logistic regression analysis and age‐adjusted ANOVA for repeated measurements.

The clinicodemographic features of the study population are reported elsewhere.^2^ In our cohort, cOH occurred in 19% (n = 32) of patients and tOH in 24% (n = 41).

**FIG. 1:**
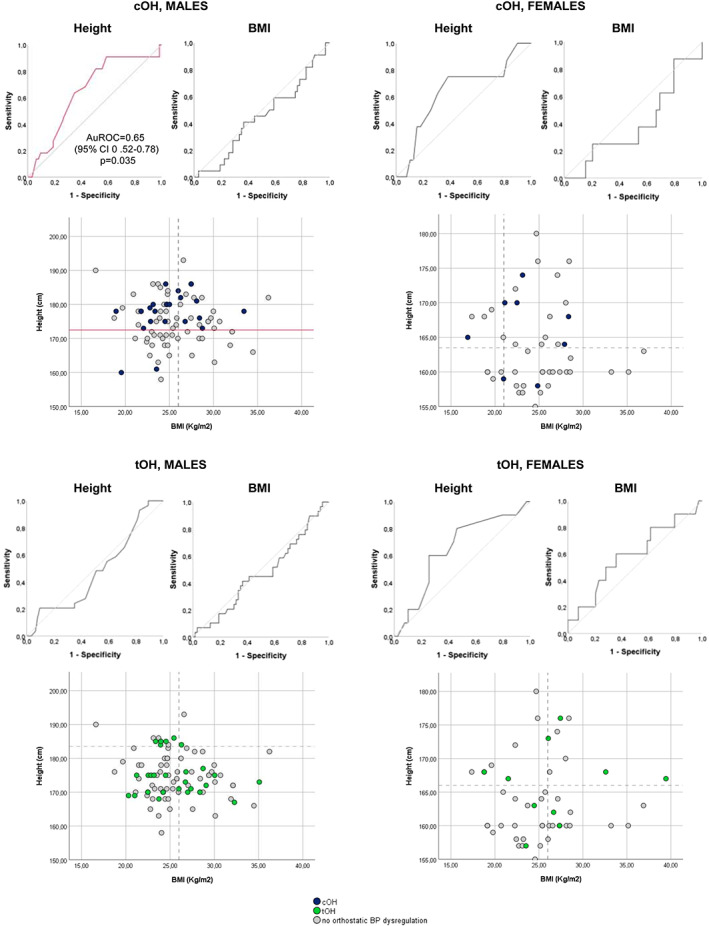
Receiver operating characteristic (ROC) curves and scatterplots of height and body mass index (BMI) in male and female patients with Parkinson's disease (PD) with laboratory‐confirmed classic orthostatic hypotension (cOH) and transient OH (tOH) versus no orthostatic blood pressure dysregulation. The red line indicates the significant associations detected at the ROC curve analysis and related height cutoff values distinguishing male patients with PD with cOH versus no orthostatic BP dysregulation, calculated by applying the Youden index to the coordinates of the area under the ROC curves (AuROC). Nonsignificant ROC curves and cutoff values are reported with gray lines. CI: confidence interval. [Color figure can be viewed at wileyonlinelibrary.com]

BMI did not differ between patients with either cOH (*P* = 0.270) or tOH (*P* = 0.798) compared with those without OH (Fig. 1).

The ROC curve analysis excluded any differences in height among female patients with or without OH, but pinpointed a positive association between cOH and taller stature in male patients (Fig. 1). Male patients with cOH did not otherwise differ for any other clinicodemographic characteristic from those with tOH or no OH. The Youden index identified a height cutoff of ≥172.5 cm for predicting cOH in male patients with PD (Fig. 1). Both univariate and age‐adjusted logistic regression analysis confirmed a negative association between cOH and shorter stature in males (odds ratio = 0.14 [95% confidence interval, 0.03–0.66]; *P* = 0.013), despite higher, yet not significant after Benjamini–Hochberg correction, frequencies of cardiovascular comorbidities and use of antihypertensive medications (Supporting Information Table S1).

At hemodynamic monitoring, shorter patients showed an average systolic BP increase after 3 minutes on standing, while patients ≥172.5 cm tall had a decrease (*P* = 0.030; Supporting Information Fig. S1). The remaining cardiovascular autonomic function indices did not differ across the height groups (Supporting Information Fig. S1).

Pilot studies in Asian PD populations suggested an association between lower BMI and cOH.^4^, ^5^, ^6^ Here we did not observe any difference in BMI across male or female patients with PD with either cOH, tOH or no OH. This inconsistency possibly reflects ethnic and morphometric differences between European and Asian natives.

Elderly, otherwise healthy, shorter subjects show higher BP values compared with taller subjects, potentially reflecting underlying hydrostatic mechanisms.^7^ The fact that cardiovascular autonomic function indices other than cOH were equally impaired in shorter and taller patients suggests that analogous, non‐neurogenic mechanisms may prevent shorter individuals with PD from developing clinically relevant BP declines on standing.

Identifying individual OH risk factors may optimize screening measures for this frequently overlooked condition.

## Supporting information


**Figure S1** Analysis of variance of supine to standing HR, systolic BP and diastolic BP of male PD patients with cOH or without any kind of orthostatic blood pressure dysregulation with height < 172.5 cm compared to ≥172.5 cm. Abbreviations: BP = blood pressure; HR = heart rate.

**Table S1** Univariate analysis of clinical‐demographic features and CAFTs in male PD patients with cOH or without any kind of orthostatic blood pressure dysregulation with height < 172.5 cm compared to height ≥ 172.5 cm.

## Data Availability

Due to the retrospective nature of the study, no ethic approval or written informed consent was due. We performed the study in accordance with the Declaration of Helsinki and the current European data protection regulation. The first and last author take responsibility for the integrity of the data and the accuracy of the data analysis. The authors have full access to all of the data, have the right to publish any and all data separate and apart from any sponsor, to obtain independent statistical analyses of the data. We agree to share any data not published within this article upon reasonable request from any qualified investigator.
