# Supplementary material for: Orthostatic Hypotension in Parkinson's Disease: Do Height and Weight Matter?
Source: Mov Disord. 2021 Aug 23;36(11):2703–5. doi: 10.1002/mds.28768 (PMC9292422; doi:10.1002/mds.28768)
Supplement: Supplementary file 2 — Table S1 Univariate analysis of clinical‐demographic features and CAFTs in male PD patients with cOH or without any kind of orthostatic blood pressure dysregulation with height < 172.5 cm compared to height ≥ 172.5 cm. [file MDS-36-2703-s001.docx]

**Supplementary Table 1**

**Univariate analysis of clinical-demographic features and CAFTs in male PD patients with cOH or without any kind of orthostatic blood pressure dysregulation with height < 172.5 cm compared to height ≥ 172.5 cm**

|  | **Height < 172.5 cm**  **(n = 28)** | **Height ≥ 172.5 cm**  **(n = 57)** | **p** |
| --- | --- | --- | --- |
| **Demographic characteristics** | | | |
| BMI (Kg/m^2^) | 24.54 [23.09; 28.17] | 24.81 [23.25; 27.62] | 0.911 |
| underweight | 0 (0) | 1 (2) | 0.861^1^ |
| norm-weight | 17 (61) | 29 (51) |  |
| overweight | 6 (21) | 23 (40) |  |
| obese | 5 (18) | 4 (7) |  |
| Age, years | 74 [69; 78] | 70 [67; 75] | 0.037 |
| **PD-related characteristics** | | | |
| Disease duration, months | 8 [4; 11] | 6 [3; 8] | 0.164 |
| Hoehn & Yahr stage | 2 [2; 3] | 2 [2; 3] | 0.389 |
| LEDD, mg/day | 763 [338; 1015] | 599 [255; 936] | 0.178 |
| **Comorbidities** | | | |
| Cardiovascular comorbidities (structural, ischemic or arrhythmic) | 14 (50) | 12 (21) | 0.006 |
| Anti-hypotensive drugs | 2 (7) | 6 (11) | 1 |
| Anti-hypertensive drugs | 15 (54) | 16 (28) | 0.022 |
| **CAFTs** | | | |
| Pathological age-adjusted Valsalva ratio, n=65 | 14 (64) | 22 (51) | 0.338 |
| Pathological age-adjusted deep breathing ratio, n=72 | 16 (67) | 33 (69) | 0.858 |
| Missing Valsalva phase IV BP overshoot, n=63 | 19 (91) | 38 (91) | 1 |

We reported qualitative variables by frequency (percentage) and compared them with the Pearson’s Χ2 test (or Fisher’s exact test, where appropriate). We summarized quantitative variables by median [1^st^ quartile; 3^rd^ quartile] and tested for differences between groups with the T test or the Mann–Whitney U test, according to their distribution in Shapiro-Wilk test. We applied a Benjamini-Hochberg correction to multiple comparison.

^1^ due to the small sample size of the underweight group, underweight and norm-weight patients are computed together. The p value refers to the comparison between under-/norm-weight, overweight and obese subgroups.

Abbreviations: BP = blood pressure; BMI = body mass index; CAFTs = cardiovascular autonomic function tests; LEDD = L-dopa equivalent daily dose.
